# Impact of Physical Activity on the Self-Perceived Quality of Life in Non-Frail Older Adults

**DOI:** 10.14740/jocmr2021w

**Published:** 2015-06-09

**Authors:** Ulla Svantesson, Janelle Jones, Kristin Wolbert, Marie Alricsson

**Affiliations:** a University of Gothenburg, The Sahlgrenska Academy, Institute of Neuroscience & Physiotherapy, SE 405 30 Goteborg, Sweden; b Johns Hopkins University School of Nursing, 525 North Wolfe Street, Baltimore, MD 21205, USA; c North Carolina Agricultural & Technical State University School of Nursing, 1601 E. Market Street, Greensboro, NC 27411, USA; d Department of Health Science, Swedish Winter Sports Research Centre, Mid Sweden University, SE 83125 Ostersund, Sweden

**Keywords:** Elderly, Healthy aging, Physical performance, Public health

## Abstract

As the population of older adults increases, healthy aging has become a global public health issue. Physical activity can help older adults reclaim or maintain a healthy aging process. The purpose of this paper is to investigate the relationship between physical activity, physical performance, quality of life and cognition in non-frail adults aged 65 and older. English articles in peer-reviewed journals about healthy, non-frail adults aged 65 and older were included in the present review. Additionally, articles were obtained from reviewing the reference lists of the aforementioned articles. Research proves an overwhelmingly positive correlation between physical activity and the reduction of preventable chronic illnesses, lower healthcare costs, improved cognition, improved muscle function, decreased fear of falling, and thereby, inevitably, an increased self-perceived quality of life. There is research evidence on healthy aging and the effect of physical activity, which could be of importance in a public health perspective.

## Introduction

According to the World Health Organization, being physically inactive is a leading cause of mortality [1]. As humans, age muscle strength is decreased due to skeletal muscle atrophy. This leads to decreased mobility, an increase with assistance of activities of daily living and fear of falling and hip fractures [2, 3]. As a result of aging, there is an inevitable decrease in all body systems leading to “weakness, fatigue, and slowing of movement” which aids in the possibility of an older adult needing assistance to complete their activities of daily living [4]. When physical activity is continued throughout the life, the occurrence and frequency of different chronic illnesses, both physical and mental, is decreased [2].

Healthy aging, also known as successful aging, if left undefined, can be viewed very subjectively. It can be defined as simply as being without the presence of illness, or the absence of decreased cognition in old age. However, a more multidimensional approach of defining healthy aging is presented by the World Health Organization, as obtaining or maintaining “physical, intellectual, emotional, social, vocational and spiritual functioning” at the best possible self-perceived level for aging adults [5].

Physical activity has many different meanings (Table 1) [5-9]. For this review, we will use the definition which Koeneman et al used which states that physical activity is unstructured and integrated into everyday life and exercise in a prearranged, deliberate, and repetitive manner [6].

**Table 1:** Key Terms and Definitions

Key term	Definition
Physical activity	“Any bodily movement produced by skeletal muscles that result in energy expenditure’’ above resting (basal) levels. Physical activity broadly encompasses exercise, sports, and physical activities done as part of daily living, occupation, leisure, and active transportation” (garber). It is an unstructured and integrated into everyday life and exercise in a prearranged, deliberate, and repetitive manner [6].
Exercise	“Physical activity that is planned, structured, and repetitive and (that) has as a final or intermediate objective the improvement or maintenance of physical fitness” [7].
Physical fitness	“The ability to carry out daily tasks with vigor and alertness, without undue fatigue and with ample energy to enjoy (leisure) pursuits and to meet unforeseen emergencies. Physical fitness is operationalized as “(a set of) measurable health and skill-related attributes” that include cardiorespiratory fitness, muscular strength and endurance, body composition and flexibility, balance, agility, reaction time and power” [7].
Energy expenditure	“The total amount of energy (gross) expended during exercise, including the resting energy expenditure (resting energy expenditure + exercise energy expenditure). Energy expenditure may be articulated in METs, kilocalories or kilojoules” [7].
MET	“An index of energy expenditure. “(A MET is) the ratio of the rate of energy expended during an activity to the rate of energy expended at rest (One) MET is the rate of energy expenditure while sitting at rest...by convention, (1 MET is equal to) an oxygen uptake of 3.5 mL/kg/min” [7].
MET-minutes	“An index of energy expenditure that quantifies the total amount of physical activity performed in a standardized manner across individuals and types of activities. Calculated as the product of the number of METs associated with one or more physical activities and the number of minutes the activities were performed (i.e., METs × minutes). Usually standardized per week or per day. Example: jogging (at about 7 METs) for 30 min on 3 days/week: 7 METs × 30 min × three times per week = 630 MET min/week” [7].
Sedentary behavior	Activity that involves little or no movement or physical activity, having an energy expenditure of about 1 - 1.5 METs. Examples are sitting, watching television, playing video games, and using a computer.
Healthy aging	As obtaining or maintaining “physical, intellectual, emotional, social, vocational and spiritual functioning” (Ng) at the best possible self-perceived level for aging adults [5].
Quality of life	Encompasses “physical and social functioning, emotional well-being, role activities, and individual health perceptions” and is measured by using different questionnaires, and is sometimes referred to as health-related quality of life [8].
Functional performance	The physical, psychological, social, occupational, and spiritual activities that people actually do in the normal course of their lives to meet basic needs, fulfill usual roles, and maintain their health and well-being” [9].

Different key terms with their definitions as expressed within this paper.

The American College of Sports Medicine (ACSM) recommends all adults over the age of 65, who have no physical activity limitations have a specific plan for physical activity which includes “aerobic, muscle-strengthening, and flexibility activities (and possibly balance exercises)”. These activities need to meet the standards of being both preventative and therapeutic [10]. Pedersen and Saltin list possible contraindications for intensity of physical activity based on different comorbidities [11]. A mixture of both moderate and vigorous exercises, or each on their own can be used to meet the activity recommendations of the ACSM, provided the “criterion for total volume of energy expended is satisfied” [7]. Some studies state there is an increased health benefit with increased physical activity [1, 7, 10-14]. The more active a person is, the greater the health benefit. Data regarding the specific quantity and quality of physical activity for the attainment of the health benefits are less clear [7].

Based on the recommendations from the ACSM and the American Heart Association (AHA), our review focuses on the following aspects of physical activity: cardio-respiratory (aerobic), muscle strengthening (resistance), flexibility and neuromotor exercises.

Aerobic physical activity is any activity that increases the heart rate due to increased demand. Continuation of this type of activity makes the entire cardio-respiratory system stronger [15]. The ACSM relates aerobic physical activity to fitness and recommends an intensity level that uses 50-85% of a person’s oxygen uptake reserve. This will include using “both moderate and vigorous exercises” [10]. This means having an intensity level between 5 and 9 if relating it to a 10-point scale. Promotion and maintenance of health begins when adapting aerobic activity for 30 min a day for at least 5 days a week or vigorous intensity for at least 20 min a day for at least 3 days a week. To meet this recommendation, a mixture of moderate and vigorous intensity can be used. All of this should be used on top of lower intensity activities of daily living that are routinely completed [10].

Muscle strength, or resistance training, needs to be at least maintained, if not increased to assist with increasing bone density and decrease the risk, or prevent osteoporosis [7]. The ACSM recommends completing 8 - 10 exercises using all major muscle groups on at least two non-consecutive days each week. Each exercise should use a weight, or resistance, which permits for each exercise to be repeated 10 - 15 times. A moderate to high level of exertion needs to be used [10] to produce benefits related to healthy aging. With consistent usage of all major muscle groups, greater flexibility should ensue.

Physical activity helps preserve bone mass, which can help decrease the risk for falls and injuries from falls by up to 35-45% [10]. Specifically neuromotor exercises (a combination of balance, coordination, gait, agility, and proprioceptive training [7]) three times a week have been proven to be valuable in preventing falls and the fear of falling [7, 10].

“Functional performance has been defined as the physical, psychological, social, occupational, and spiritual activities that people actually do in the normal course of their lives to meet basic needs, fulfill usual roles, and maintain their health and well-being” [9]. Quality of life, which encompasses “physical and social functioning, emotional well-being, role activities, and individual health perceptions” [8] is measured by using different questionnaires, and is sometimes referred to as health-related quality of life [8]. Given all these aspects are interrelated with physical activity, introducing/increasing physical activity in older adults, should be tackled in a multidimensional approach to healthy aging, rather than isolated strategies.

Pedometers are not an exact way to measure the capacity of physical activity, but they are successful tools in aiding people to increase their amount of physical activity. They can provide information about the number of steps a person takes, but not the speed a person traveled or the length of time they were moving [7].

A way to accurately track physical activity is by using an accelerometer, a small electronic device that can be worn on a belt. An accelerator can independently evaluate and calculate the physical activity level of a person. They are recommended to wear throughout a person’s physical activity [9, 16].

Researchers most commonly use the measurement of metabolic equivalent of tasks (METs) to measure the energy exerted during physical activity [17]. Current research suggests that activity levels exerting MET levels between 3 and 5 are needed to produce results related to healthy aging in older adults [7, 16].

The purpose of the present literature review was to examine the impact of physical activity on health-related quality of life in non-frail older adults.

## The Literature Search and Inclusion Criteria

**Figure 1:**
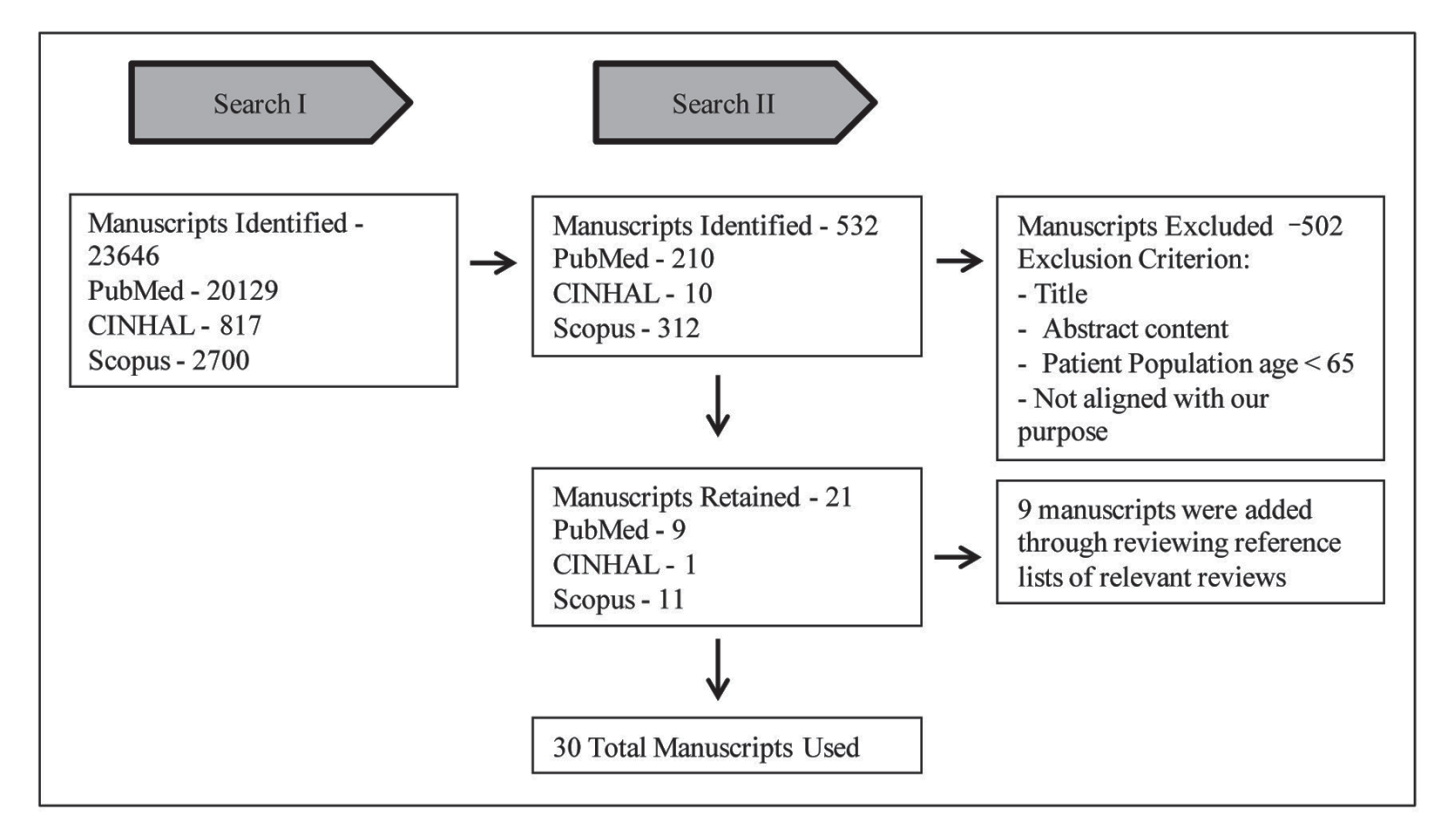
Flowchart of manuscript selection process for review.

PubMed, CINHAL and Scopus were the databases used for our research (Fig. 1). Our criteria included: English articles in peer-reviewed journals about healthy, non-frail adults aged 65 and older. Our mesh terms included: humans, healthy, physical activity, elderly/older adults, cognition/brain health, quality of life and muscle strength. Additionally, we obtained articles from reviewing the reference lists of the aforementioned articles.

Search I was conducted on July 31, 2013 with initial search with mesh terms noted above.

Search II was conducted on August 01, 2013 with secondary search with included mesh terms: humans and healthy, and we placed quotation marks around all terms to more efficiently capture the manuscripts that best the purpose of this review. After reading the 30 remaining articles, 12 articles were finally included in the systematic review.

## Findings

Summary of information from literature review search is presented in Table 2 [9, 14, 18-27].

**Table 2:** Summary of Information From Literature Review Search

Article No.	Authors, publication date, study title, journal	Data collection procedures	Study population	Major findings
1	Benedict et al [18] 2013 Association between physical activity and brain health in older adults Neurobiol Aging	MRI imaging, self-report physical activity	331 cognitively healthy elderly > 75 years old	A positive association between level of physical activity and mini-mental state examination score, a negative association between physical activity and time to complete Trail-making B test, and the level of physical activity correlated with total brain volume and white matter volume. Physical activity is an important part of lifestyle to maintain healthy brain.
2	Geirsdottir et al [19] 2012 Physical function predicts improvement in quality of life in elderly Icelanders after 12 weeks of resistance exercise J Nutr Health Aging	Dual energy X-ray absorptiometer, TUG test and 6MW, hydraulic hand dynamometer, isokinetic dynamometer, and HRQQL questionnaire	238 participants aged ≥ 65	Changes in the 6-minute walk for distance significantly improve lean mass, muscle strength, physical function, and health related quality of life (HRQL). The improvements in physical function predict improvements in HRQL.
3	Ho et al [14] 2011 The effects of physical activity, education, and body mass index on the aging brain Hum Brain Mapp	MRI, BMI, Minnesota Leisure-Time Activities questionnaire, human brain mapping	226 with mean age of 77.9 ± 3.6 SD	Physical activity is associated with about 2-2.5% greater average white tissue volume. Increased physical activity is associated with greater average brain tissue volumes.
4	Aoyagi et al [20] 2010 Habitual physical activity in the elderly: The Nakanojo study Geriatrics & Gerontology	Physical activity questionnaire, uniaxial pedometer/accelerometer	5,000 older adults over 65 (one-tenth randomly selected to assess physical activity)	20 min/day of moderate walking along with over 60 min/day light activity leads to better physical health. A smaller amount of deliberate physical activity is associated with increased mental health.
5	Muscari et al [21] 2010 Chronic endurance exercise training prevents aging-related cognitive decline in healthy older adults: A randomized controlled study Int J Geriatr Psychiatry	3 hourly sessions/week for 12 months, baseline and post-intervention assessments of MMSE, subsub-maximal cycle ergometer test, selected anthropometric measurements, and blood drawing for laboratory determinations, PASE questionnaire	120 healthy subjects between 65 and 74 years old	Cognitive decline may be reduced with the use of endurance exercise training as assessed by the mini-mental state examination.
6	Fitzpatrick et al [22] 2007 Associations of gait speed and other measures of physical function with cognition in a healthy cohort of elderly persons J Gerontology	TICS questionnaire, 3MSE, 14 neuropsychiatric tests, anthropometric measures of height and weight, self-reports, 15-feet timed walk (regular pace and fast pace)	3,035 healthy mobile adults ≥ 75 years old	Significant association between lower modified mini mental state examination and time to walk 15 feet at rapid pace.
7	Goodpaster et al [23] 2006 The loss of skeletal muscle strength, mass, and quality in older adults: The health, aging and body composition study J Gerontology	Isokinetic dynamometry, dual-energy X-ray absorptiometry and computed tomography	3,075 men and women aged 70 - 79 years	Maintaining or gaining muscle mass does not prevent aging-associated declines in muscle strength.
8	Karinkanta et al [24] 2005 Factors predicting dynamic balance and quality of life in home-dwelling elderly women Gerontology	Rand-36 questionnaire, figure-of-eight running test, leg press dynamometer, ground reaction forces (GRF) with a force platform, a sit-to-stand and a step-on-a-stair, and simple reaction time paradigm, employing a random light or sound stimulus and a finger pushbutton as the response to the stimulus	153 women aged 70 - 78 years old	Dynamic balance is one of the most important determinants to protect independency later in life and good indicator of quality of life.
9	Rosano et al [25] 2005 Association between physical and cognitive function in healthy elderly: The health, aging and body composition study Neuroepidemiology	3MS, DSST, gait speed was measured (m/s) during two 6-m walks, both performed at usual or normal pace, NW test required the participant to walk between lines of colored tape on the floor, 20 cm apart	3,075 nondisabled men and women aged 70 - 79 years	There is a significant association between physical activity and cognitive function.
10	Brouwer et al [26] 2004 Physical function and health status among seniors with and without a fear of falling Gerontology	SF-36, ABC scale, Human Activity Profile questionnaire, limits of stability, walking speed, lower extremity muscle strength	25 older adults in fear of falling group and 25 older adults in control group	Fear of falling is associated with lower physical function.
11	van Gelder et al [27] 2004 Physical activity in relation to cognitive decline in elderly men: The FINE study Neurology	Physical activity self-report questionnaire, MMSE, and questionnaires for demographic, lifestyle and other information	295 older men	Physical activities of medium-low intensity are associated with a decrease in cognitive decline more so than activities of the lower intensity.
12	Muraki et al [9] 2001 A preliminary investigation to explore the effects of daytime physical activity patterns on health-related QOL in healthy community-dwelling elderly subjects Phys Occup Ther Geriatr	Mini-Motionlagger Actigraph, Zung Self-Rating Depression Scale, Life Satisfaction Index-Z, and Visual Analogue Scale of Happiness	69 elders	Gender is not factor, but marital status is in the difference in the correlation between physical activity and quality of life. Quality of life may differ by gender.

**Figure 2:**
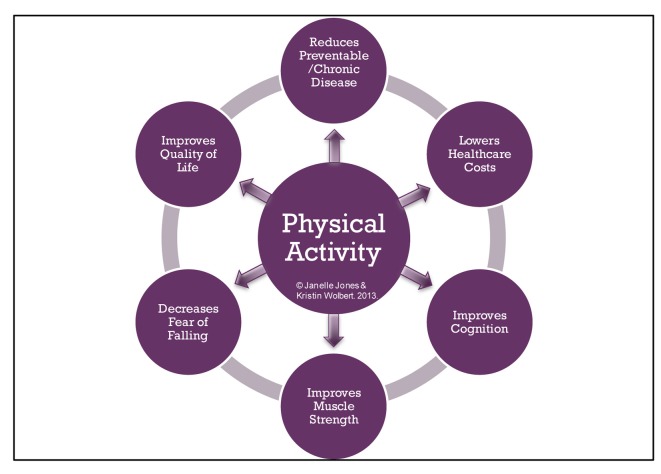
Relationship between physical activity and components of healthy aging. Physical activity has a positive correlation with the reduction of preventable chronic illnesses, lowering healthcare costs, improving cognition, improving muscle function, decreasing fear of falling, and increasing self-perceived quality of life.

The information obtained in our research all showed a positive correlation between physical activity and the reduction of preventable chronic illnesses, lower healthcare costs, improved cognition, improved muscle function, decreased fear of falling, and thereby, inevitably, an increased self-perceived quality of life (Fig. 2).

## Literature Review and Discussion

### General benefits of physical activity

The purpose of this review was to investigate the relationship between physical activity and healthy aging, in non-frail adults aged 65 and older. Our definition of non-frail adults includes older adults with no assistive devices, canes exempt, and no diseases that cause physical or mental impairment. We hypothesized that there would be a positive correlation between physical activity and healthy aging and research has proven empirical evidence in positively affirming our hypothesis [1, 7, 10-14].

In older adults, physical activity has a myriad of positive interrelated physical, psychosocial and economic benefits [28]. They range from decreased fear of falling to maintained or increased cognitive function, to maintained or increased muscle function, to an increased quality of life related to maintaining or increasing autonomy in older age. While there are risks of injury while participating in physical activity, the benefits of increased cardiorespiratory health, cognition, muscle strength, flexibility, and balance are much greater [1, 7, 10-14]. Physical activity empowers older adults to engage in positive strategies to maintain or increase their health while aging. Physical activity also promotes autonomy with increased functional performance, to allow for a more dynamic lifestyle [9].

Although there is increasing research-based evidence of the benefits of physical activity for healthy aging [1, 7, 10-14], there are still numerous barriers of engagement in physical activity for older adults. Culos-Reed states that some barriers may include: the fear of injury, social isolation, lack of knowledge, lack of availability and access to physical activity programs and a lack of motivation [28]. To mitigate against these barriers, it may prove beneficial to provide health education about the benefits of physical activity to the entire community, especially older adults. Increased health literacy can provide a platform for the demystification of the fear of injuries. Additionally, increased health literacy may foster an increased demand for the organization of safe, social physical activities for older adults (i.e. local mall walking programs).

Researchers have estimated that by the year 2030, 14% of the population will be comprised older adults [14] aged 65 and older. Additionally, by the year 2050, the population of older adults will increase to 20% [29]. Because of this influx of older adults, the discussion and implementation of the benefits of healthy aging are necessary, relevant and important to the global community. In older adults, healthy aging may be the difference between maintaining an autonomous, highly functional lifestyle, well integrated in society versus a dependent, low functional lifestyle, managed by others. Management by others may include dependent care from: hospitals, nursing homes, older adult daycare centers or other organized institutions. Management of older adults commonly extends to family members and spouses.

In an effort to maintain a global society of older adults who have a maximum perceived quality of life, the entire lifespan must be considered. Healthy aging is an important strategy to help maintain or increase viable societies filled with independent older adults, free of unnecessary sedentary lifestyle illnesses and their related unnecessary medical costs. Because of the vast benefits physical activity provides with healthy aging, physical activity is recommended for execution in the daily lives of all capable older adults, under the supervision of a healthcare professional [10].

### Reduces preventable, chronic illnesses

Physical activity, exerted moderately and with daily consistency can be used as a preventative measure to ward off preventable, chronic illnesses, decrease the occurrence of common diseases found in older adults, such as Parkinson’s disease and dementia, and decrease mortality [12]. The implementation of physical activity as part of the treatment and prevention of various chronic conditions should be a priority and implemented extensively [10].

According to Pedersen and Saltin, there is substantial understanding over the past decades concerning the importance of physical activity and management of chronic illnesses, besides the disorders of the musculoskeletal system [11]. Physical activity in short bursts, to break up sedentary behaviors, can decrease the risks for: certain cancers (colon and breast), type 2 diabetes mellitus, hypertension, coronary heart disease (CHD), obesity, stroke, osteoporosis [13, 14], cognitive impairment and disability, anxiety and depression [7, 10]. Garber also concludes that physical activity helps enhance insulin sensitivity, lipoprotein lipase activity, C-reactive protein and other chronic disease biomarkers such as blood glucose, insulin, and lipoproteins [7]. A reoccurring theme within all of the research suggested that physical activity also helped to improve weight management.

### Lowers healthcare costs

The older adult population is the least active and has the highest healthcare costs. Physical activity is a no/low cost option of helping older adults maintain independent living, enhancing their satisfaction of autonomy and self-efficacy [30] while participating in healthy aging. By increasing physical activity (above 3 METs) among the older adult population, their health care costs could be reduced [10] along with the risk for dementia and other neurodegenerative disorders [14]. Besides reducing healthcare costs, the overall health and quality of life of an older adult can be improved by increasing physical activity [13].

### Prevents cognitive decline

Researchers have proven through large observational [22], clinical and experimental [23] studies that there is a positively inverse correlation between the increase of physical activity and the decrease of cognitive decline [7, 12-14, 18, 20-22, 25]. Theories surrounding the exact link between physical activity and increased cognition are still emerging. Some theories suggest that the enhancement of sleep and the reduction of stress [2] is a direct cause of physical activity which can affect increased cognition. Physical activity can potentially decrease cognitive decline as it increases cerebral blood flow [12] which increases brain-derived neurotropic factor (BDNF) and insulin-like growth factor-1 (IGF-1), which helps stimulate neurogenesis in the hippocampus [14, 21, 27]. Neurogenesis helps to preserve hippocampal gray matter volume which is related to the increase of memory functions [18], thereby decreasing cognitive decline.

The brain is a very complex organ, and pinpointing the specific cognitive abilities that can best be affected by physical activity has proven problematic for some researchers. However, task oriented cognitive functioning has been classified into the following categories: reaction time, spatial, controlled and executive functioning [12, 16]. Kimura stated that a recent meta-analysis proved that the cognitive aspect most positively affected by physical activity is executive functioning [12, 16]. Another study has linked increased aerobic activity to increased spatial memory functioning [14]. Although cognitive diseases such as Alzheimer’s and dementia have been linked to genetics [18], research has proven that the incidences of these diseases, especially dementia, can be drastically reduced by physical activity [7]. One year of continuous, moderate-intensity aerobic exercise is recommended in association with preserving cognitive function [18].

### Decreases fear of falling

According to Robinovitch, inadvertent injuries in older adults are most commonly caused by falls. Inadvertent injuries, from falls, account for “90% of the hip and wrist fractures and 60% of the head injuries” [31] of all older adults. On average, 50% of older adults living in a continuous care facility fall every year. Whereas, 30% of older adults, who live at home, fall every year. Balance is a significant aspect of preventing falls among older adults. Falls can be improved when older adults use balance and strength training together [24, 31]. These can also strengthen bones and allow an older adult the freedom to live without a fear of having a serious injury if they should fall.

Fear of falling is associated with lower physical activity and this is evident from moving slower, decreased muscle strength, and not seeing themselves as having a high level of wellbeing. Having a fear of falling can indicate deterioration in overall physical activity. It can also jeopardize independence and quality of life [26]. This should be improved with increased physical activity which includes flexibility training and neuromotor exercises, along with aerobic and muscle strengthening exercises.

### Improves muscle strength

Loss of muscle strength and mass is inevitable along with the decrease of type 2 (fast twitch) muscle fiber size [4]. This muscle weakness is more evident in older adults who are physically inactive, or have a lower activity level. This leads to the inability to complete activities of daily living and a decrease in quality of life and an increase in their healthcare costs [19]. Muscle weakness is also a risk factor for falling and mortality in older adults [23].

Since losing muscle mass and strength is an inevitable part of aging, there needs to be a way to decrease the rate of decline. Physical activity in the form of resistance training is a documented way of slowing down this process [4]. Geirsdottir discovered that “lean mass, muscle strength, physical function and HRQL” all improved after 12 weeks of resistance training [19]. Karinkanta provided evidence that muscle strength correlates to walking speed and by increasing muscle strength, an older person’s balance improves. Moderate physical activity done on a consistent basis leads to a reduced number of hip fractures in women by up to 6% [24].

### Improves quality of life

Quality of life can be assessed by many different facets of a physically active lifestyle. The more physically activity an older adult is directly correlates to having a better quality of life. This is due to the increase in muscle performance, balance, cognition health, and ability to be independent. There is a direct correlation between muscle strength and quality of life (SF-36) scores. There is a link between the time an older adult can walk 6 m and run a figure eight with how their quality of life is. The stronger a person’s muscles are the faster they can walk and complete their daily activities. All of this increases a person’s quality of life because they can live a more independent life [19, 24].

One of the most important findings was balance. It is an extremely good indicator of an older person’s quality of life because it is incorporated to every aspect of daily life. This is an early indicator of decrease quality of life and increase in fear of falling. Balance allows for a person to live an independent lifestyle longer [24]. When combining muscle strength and balance, a person’s quality of life is increased because they are able to carry out activities of daily living and not have a fear of falling or having an injury. If a person was to fall, they should have a lower risk of injury because the physical activity they have been doing assists with having strong bones, strong minds, and strong muscles. All of these aspects, along with having a strong mind, lead to independence and a higher quality of life.

### Activity plan

According to guidelines from the ACSM and the AHA, areas of emphasis in promoting physical activity in older adults include: reducing sedentary behavior (lower risks of cardiovascular disease have been observed with just 45 - 75 min of walking per week (4.0)), increasing moderate activity and giving less emphasis to attaining high levels of activity and taking a gradual or stepwise approach. When developing their own activity plan, older adults should consult with their “healthcare provider or fitness professional, so as to take advantage of expertise and resources on physical activity and injury prevention” [10]. An activity plan allows for accountability and individualization for the older adult. As physical status changes, the plan can be altered to show improvements have been made and confidence levels rising. Older adults are able to achieve increasing levels of physical activity as their skills improve and they gain experience. It also gives control to the older adult and allows them to have physical activity they are comfortable with and stay in their comfort zone. Given the breadth and strength of the evidence, physical activity should be one of the highest priorities for preventing and treating disease and disablement in older adults [10].

## Conclusion

Physical activity has a positive correlation with healthy aging in older adults. Therefore, all capable older adults should be engaging in physical activity for at least 30 min a day for 5 days per week. There is research evidence on healthy aging and the effect of physical activity, which could be of importance in a public health perspective.
